# Salivary microbiome composition changes after bariatric surgery

**DOI:** 10.1038/s41598-020-76991-6

**Published:** 2020-11-18

**Authors:** Mária Džunková, Róbert Lipták, Barbora Vlková, Roman Gardlík, Michal Čierny, Andrés Moya, Peter Celec

**Affiliations:** 1 Department of Energy Joint Genome Institute, Lawrence Berkeley National Laboratory, Berkeley, CA USA; 2 Institute of Molecular Biomedicine, Faculty of Medicine, Comenius University, Bratislava, Slovakia; 3 Department of Bariatric Surgery, Břeclav Hospital, Břeclav, Czech Republic; 4 Department of Genomics and Health, Foundation for the Promotion of Sanitary and Biomedical Research of Valencian Community (FISABIO-Public Health), Valencia, Spain; 5 CIBER in Epidemiology and Public Health (CIBEResp), Madrid, Spain; 6 Institute for Integrative Systems Biology (I2SysBio), University of Valencia and Spanish National Research Council (CSIC), Valencia, Spain

**Keywords:** Endocrine system and metabolic diseases, Microbiology, Microbial communities

## Abstract

Recent studies show that the salivary microbiome in subjects with obesity differ from those without obesity, but the mechanism of interaction between the salivary microbiome composition and body weight is unclear. Herein we investigate this relation by analyzing saliva samples from 35 adult patients with obesity undergoing bariatric surgery. Our aim was to describe salivary microbiome changes during body weight loss on an individual-specific level, and to elucidate the effect of bariatric surgery on the salivary microbiome which has not been studied before. Analysis of samples collected before and 1 day after surgery, as well as 3 and 12 months after surgery, showed that the salivary microbiome changed in all study participants, but these changes were heterogeneous. In the majority of participants proportions of *Gemella* species*, Granulicatella elegans, Porphyromonas pasteri, Prevotella nanceiensis* and *Streptococcus oralis* decreased, while *Veillonella* species*, Megasphaera micronuciformis* and *Prevotella saliva* increased. Nevertheless, we found participants deviating from this general trend which suggests that a variety of individual-specific factors influence the salivary microbiome composition more effectively than the body weight dynamics alone. The observed microbiome alternations could be related to dietary changes. Therefore, further studies should focus on association with altered taste preferences and potential oral health consequences.

## Introduction

Obesity is a serious public health problem, as it is linked to increased risk of cardiovascular diseases and type 2 diabetes^1^. Worldwide, more than one third of adults have a body-mass index (BMI) of > 25 kg/m^2^, which classifies them overweight or suffering from obesity^2^. Since the discovery of the contribution of gut microbiome composition to the obesity risk^3^, the role of the gut microbiome in metabolism has been studied extensively^4^. It has been found that some of the common gut microbes contribute to obesity development by providing an extra energy source, or by enhancing gut-brain-mediated fat intake and hunger signalling^5,6^.

Recent comparative studies show that the microbiome differences between patients with obesity and lean volunteers are not limited to the gut, but are also apparent in the salivary microbiome^7–12^. One of the possible explanations for the differences in salivary microbiome composition is hyperglycemia due to obesity-related diabetes which results in high salivary glucose and acidification of the oral environment promoting growth of obesity-associated bacterial species^8^. The relationship, however, might also have the opposite causation: altered salivary microbiome can potentially influence taste preferences, quantity and quality of food consumption and liquid intake^13^.

Two previous studies report that the salivary microbiome of patients with obesity is dominated by *Streptococcus* species with a great potential for carbohydrate metabolism^14,15^. The complete list of the obesity-associated species largely differs between studies, which is not uncommon for human microbiome studies in which “diseased” and “healthy” volunteers are compared as cohorts^16^. Nevertheless, a significant decrease of BMI in patients with obesity creates a unique opportunity to study the changes of oral microbiome composition on intra-individual level, taking into account the uniqueness of microbiome of each participant and, thus, providing more accurate results enabling a correct interpretation^17^.

Bariatric surgery represents one of the most effective ways of reducing body weight. It directly limits the intake of food or interferes with its digestion^18^. Even though the surgical procedure is well-defined, the mechanism of action on a systemic level remains unclear^19^. It is well accepted that bariatric surgery is the treatment of choice not only for obesity, but also for obesity-associated type 2 diabetes^20^. Studies focused on the gut microbiome reported significant changes after different types of bariatric surgery, such as laparoscopic sleeve gastrectomy^21^, vertical banded gastroplasty (VBG)^22^, Roux-en-Y gastric bypass (RYGB) and laparoscopic adjustable gastric banding (LAGB)^23^. The mechanism behind this phenomenon might include alteration in gut hormone levels after bariatric surgery, including GLP-1, adiponectin, ghrelin and leptin. This might also influence the amount of food consumed and eventually the composition of gut microbiome^24,25^. It was found that the gut microbiome changes after the bariatric surgery^22,26^, but its relation to the salivary microbiome composition has not been studied yet.

In the present study we describe changes of the salivary microbiome composition in relation to BMI by analyzing saliva samples collected before and one day after bariatric surgery, as well as three months and one year after surgery. We aimed to identify bacterial species related to decreasing BMI and assess the effect of subject-specificity on the microbiome changes observed after bariatric surgery.

## Results

Patients with obesity (n = 35; 17 females and 18 males) with an average age of 48 ± 9 years participated in the study. Four different types of bariatric surgery were used: sleeve gastrectomy (n = 3), Roux-en-Y gastric bypass (n = 5), Omega loop gastric bypass (n = 7) and laparoscopic gastric plication (n = 20, Supplementary Table S1). During the laparoscopic gastric plication and sleeve gastrectomy the volume of the stomach is reduced in a similar way—either by folding of the stomach or removal of part of the stomach. Roux-en-Y gastric bypass and Omega loop gastric bypass are similar surgeries which involve division of the stomach in two unequal parts and the distal small intestine is connected to the smaller part of the divided stomach. The salivary microbiome composition of the patients has been assessed using 16S rRNA gene amplicon sequencing of their unstimulated saliva samples which resulted in 16,639 operational taxonomic units (OTU, Supplementary Fig. S1).

### Hospital stay

**Figure 1:**
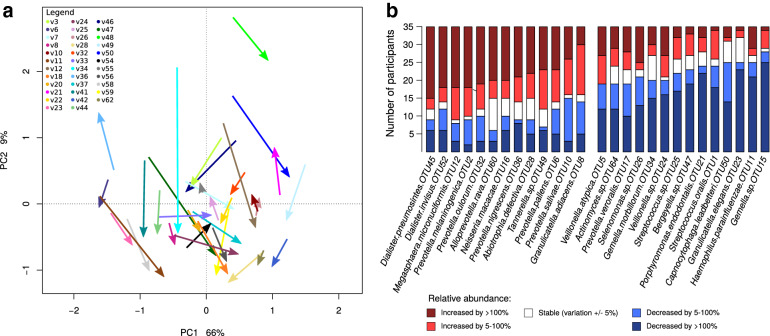
Salivary microbiome composition before and 1 day after the surgery. (**a**) Ordination of saliva samples collected before and after surgery using principal component analysis (PCA) performed on log2 transformed data. The arrows start at coordinates of the samples collected at the former time-point and they end at the coordinates of samples collected at the latter time-point. (**b**) Barplots showing the number of patients in which the relative abundance of a given OTU increased or decreased 1 day after surgery.

Analysis of the samples collected before and after the surgery performed on the whole cohort level revealed large shifts in the salivary microbiome composition (Fig. 1a). Comparison of the proportion of the individual OTUs before and one day after the surgery performed on intra-individual level revealed heterogeneity of these changes, e.g. the proportions of *Gemella* sp. OTU15 decreased after the surgery more than two times in 25 out of 35 patients, however, there were still 6 patients in which *Gemella* sp. OTU15 proportion slightly increased after the surgery (Fig. 1b). Given that no decrease of the BMI can occur immediately after a bariatric surgery, the comparison before and one day after surgery does not serve for assessing the relationship between salivary microbiome and BMI, but provides important insights into possible intra-individual variations of salivary microbiome composition after environmental stress caused by bariatric surgery and associated hospital stay.

### Microbiome changes after the surgery

**Figure 2:**
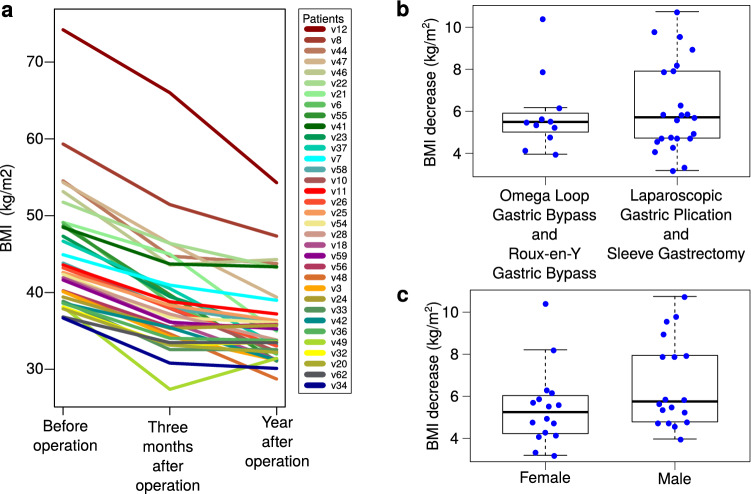
BMI of the study participants. (**a**) BMI of patients before surgery and 3 and 12 months after the surgery. (**b**) Decrease of BMI expressed as a difference of kg/m^2^ before surgery and 3 months after surgery. The patients are grouped by type of surgery (Laparoscopic Gastric Plication and Sleeve Gastrectomy n = 23, Omega Loop Gastric Bypass and Roux-en-Y Gastric Bypass n = 11). (**c**) Decrease of BMI expressed as a difference of kg/m^2^ before surgery and 3 months after surgery. Patients are grouped by sex (females n = 16, males n = 18). Boxplots in (**b**,**c**) show the median, the upper and lower quartiles.

The BMI decreased in the whole patient cohort from 44.99 ± 7.73 kg/m^2^ before surgery to 38.95 ± 7.04 kg/m^2^ 3 months after surgery (Fig. 2a). The decrease was detected in all patients (by 13.5 ± 4%), and in eight of them it decreased by more than 15%. The largest decrease was in patient v49 who reduced his BMI from 38.10 to 27.40 kg/m^2^ (by 28%). There were no significant differences in the decrease of BMI between the two different groups of bariatric surgeries (p = 0.69, t-test, Fig. 2b), nor between males and females (p = 0.095, t-test, Fig. 2c).

Decrease of BMI in the whole cohort continued and it was notable also a year after the surgery (average BMI of 35.9 ± 5.6 kg/m^2^), with the exception of five patients in which the BMI slightly increased in comparison with the 3 months after the surgery (maximum of 4 kg/m^2^ in patient 49, Fig. 2a). In other patients, the BMI 1 year after the surgery decreased by 8.9 ± 6.6% in comparison with 3 months after the surgery. None of the patients reached BMI lower than 25 kg/m^2^ (normal weight).

**Figure 3:**
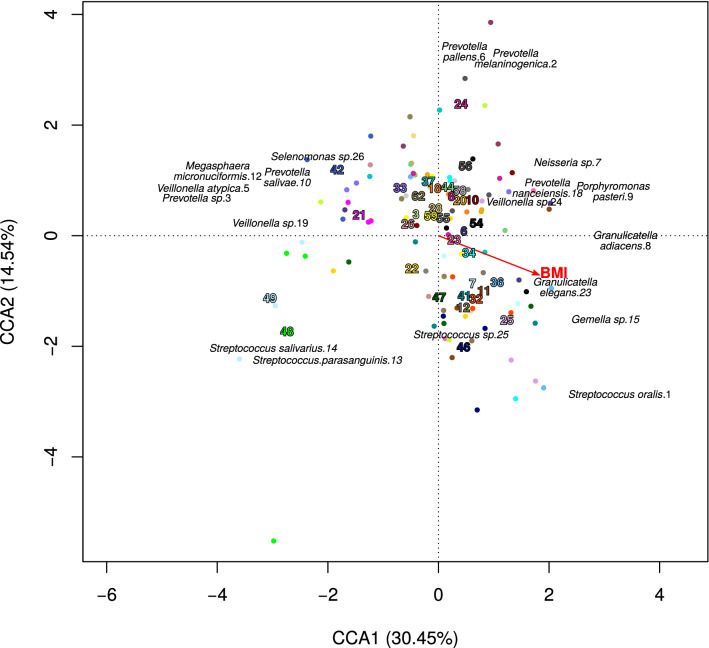
Canonical correspondence analysis. Bacterial composition of the samples from the time-points before surgery, 3 months and 12 months after the surgery, were tested for their fitting on vectors of “BMI” and “patient’s individuality”. The patient’s individuality (p < 0.001) had greater influence on ordination of samples than BMI (p < 0.01). Patient numeric IDs are placed in the centroid coordinates of each patient’s samples group which are distinguished by colour code equal to the numeric ID colour.

**Figure 4:**
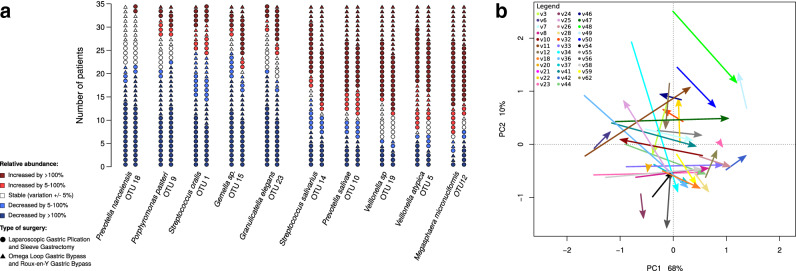
Salivary microbiome composition before and three months after the surgery. (**a**) Number of patients in which the relative abundance of a given OTU increased or decreased 1 day after surgery. Patients with different surgery types are indicated by circle or triangle as explained in the legend. (**b**) Ordination of saliva samples collected before and after surgery by principal component analysis (PCA) performed on log2 transformed data. The arrows start at coordinates of the samples collected at the former time-point and they end at the coordinates of samples collected at the latter time-point.

In the canonical correspondence analysis (CCA) we tested the ordination of salivary microbiome samples collected before surgery and three and twelve months after surgery for fitting on the vectors of “BMI” and “patients’ ID”. Vectors of *Veillonella atypica* OTU5, *Megasphaera micronuciformis* OTU12, *Prevotella* OTU10 and others appeared to be placed in the opposite direction from the BMI vector (Fig. 3). From the complete list of OTUs, only *Megasphaera micronuciformis* OTU12 had a negative correlation with BMI (Spearman correlation =  − 0.4, p-value < 0.001). In accordance with this, analysis of salivary microbiomes on individual-specific level revealed that after the significant decrease of BMI, the proportions of *Veillonella atypica* OTU5, *Veillonella* sp OTU19, *Megasphaera micronuciformis* OTU12 and *Prevotella salivae* OTU10 increased more than two times (by more than 100%) in more than half of the patients, which suggests that in general they are associated with decreasing BMI (Fig. 4a). However, the increasing proportion of these species was not ubiquitous and there were some exceptions, e.g. *Megasphaera micronuciformis* OTU12 decreased in three patients when their BMI decreased (Fig. 4a). These exceptions did not relate to the type of bariatric surgery (Fig. 4a). The patient-specific changes are supported by the PCA analysis which did not show uniform direction of microbiome changes three months after the surgery compared to the pre-surgery microbiome composition (Fig. 4b).

The vector of *S. oralis* OTU1 (the most prevalent species of the salivary microbiome before surgery), *Granulicatella elegan*s OTU23, *Gemella* sp. OTU15 and few other species appeared in the canonical correspondence ordination analysis in the same direction as BMI (Fig. 3). *S. oralis* OTU1 decreased by more than 5% in 25 out of the 35 patients (Fig. 4a) and it was the only OTU with a positive correlation with the BMI (Spearman correlation = 0.35, p-value < 0.001). The proportion of *Granulicatella elegan*s OTU23, *Porphyromonas pasteri* OTU9, *Gemella* sp. OTU15 and *Prevotella nanceiensis* OTU18 decreased more than two times (by more than 100%) in more than half of the patients (Fig. 4a). The proportion of these species increased only in a small number of patients (*Prevotella nanceiensis* OTU18 did not increase in any of the patients). These observations did not depend on the type of bariatric surgery (Fig. 4a).

### BMI vs. specificity of patient’s microbiome

**Figure 5:**
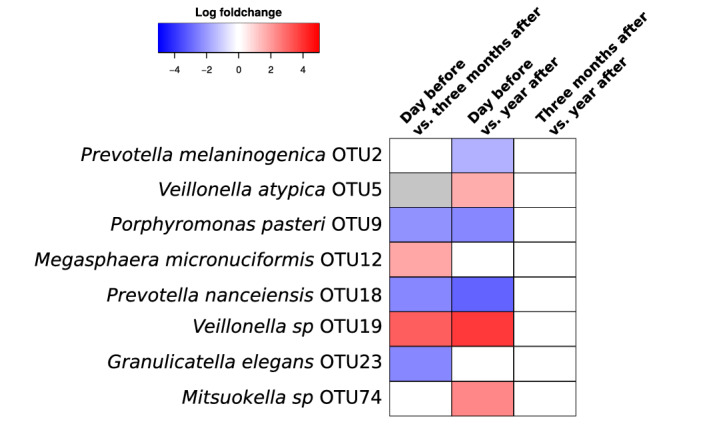
Comparison of relative abundance of bacterial OTUs between different time-points on a whole cohort level. The figure shows the results of the analysis by the “edgeR” R package. The colouring is based on log fold-change of the proportion of the bacterial species in the former time-point compared to the latter: red means an increase of the given OTU compared to the previous time-point, blue means a decrease. White colour indicates no significant change.

The results of the “envfit” test inferring the influence of environmental variables of “BMI” and “patients’ ID” on the microbial composition showed that the changes in the microbial composition are influenced more by the specificity of patient’s microbiome (p < 0.001) than by the BMI (p < 0.01, Fig. 3). Analysis on the whole-cohort level comparing paired time-points using “edgeR” package (see “Methods” section) revealed that *Veillonella atypica* OTU5, *Veillonella* sp. OTU19 and *Megasphaera micronuciformis* OTU12 were commonly increasing after surgery, and *Granulicatella elegan*s OTU23, *Porphyromonas pasteri* OTU9 and *Prevotella nanceiensis* OTU18 commonly decreasing after the surgery (Fig. 5), similarly to other analyses described above. While it was possible to identify some species commonly increasing or decreasing after the surgery, the temporal changes of salivary microbiome were not uniform. Comparison of time-points of three and twelve months resulted in an empty list of common species with a significant increase or decrease (Fig. 5), which is caused by the large heterogeneity of the long-term temporal changes in each individual microbiome. The large differences of the temporal changes among patients were confirmed also by permutational multivariate analysis of variance (PERMANOVA) which did not reveal any significant differences (p-value > 0.05) among microbial profiles of the paired time-points on the whole cohort level as a consequence of large heterogeneity among the samples.

## Discussion

Bariatric surgery affects directly the composition of the human gut microbiome by anatomical rearrangements and physiological consequences in the gastrointestinal tract^27^. Altered gut microbiome can contribute to the metabolic benefits of this intervention, as demonstrated by reduced fat deposition in experimental mice which were colonized with bacteria from stool of patients with bariatric surgery^23^. The mechanisms behind the observed change in microbiome composition after bariatric surgery are not completely understood. However, both direct and indirect effects of the surgery are considered, including weight loss, alterations in gut hormone levels or altered dietary behavior^28,29^.

In contrast to the gut microbiome, the salivary microbiome is likely not directly affected by the bariatric surgery, but its composition can be altered indirectly—salivary glucose decreases with body weight loss in diabetic patients with obesity which could enhance growth of bacterial species that prefer a less acidic oral environment^8,30^. The post-surgery period is characterized by an increased frequency of meals throughout^31^, which can result in an increase of pathogenic bacteria in the oral cavity, if effective oral hygiene is not practiced^32^. Another factor which might influence salivary microbiome composition is the gastroesophageal reflux. Although Roux-en-Y gastric bypass is considered an effective method for alleviation of gastro-esophageal reflux disease^33^, other types of bariatric surgery, such as sleeve gastrectomy, can increase the risk for gastroesophageal reflux^33^ that affects the oral pH^34^ and, thus, also the salivary microbiome^35^. Unfortunately, neither pH, nor the use of anti-reflux medications has been monitored in this study, however, we did not detect differences in salivary microbiome composition between the two different surgery types, which is in accordance with studies focused on the gut microbiome composition^22,26^. Among other factors influencing the salivary microbiome composition might be also a change in the gut-brain axis regulation. Theoretically, salivary microbiome altered by bariatric surgery could contribute to decreasing body weight by affecting the gut-brain axis regulation of body metabolism or by influencing the taste preferences and liquid intake^13,36^. In such a case, specific patterns of the salivary microbiome composition after bariatric surgery could anticipate an enhanced body weight reduction after the surgery. The compositional changes observed after the bariatric surgery in the present study were larger than the variations captured by longitudinal sampling in normal conditions^37–40^, which highlights the large impact of this surgery on the human body.

We used a variety of methods to identify bacterial species with relationship to the decreasing body weight. Some of the results obtained on the whole-cohort level, e.g. increased proportion of *Veillonella* species after the decrease of BMI, were in accordance with a recent large comparative study (n = 900) of lean volunteers and patients with obesity^11^. *Streptococcus oralis,* found to have a positive correlation with BMI in our study, has been associated with subjects with obesity in some studies^14,15^, but also with lean subjects in others^11^. *Megasphaera micronuciformis* proportion increased when the BMI of our study participant decreased, however, this species is generally not mentioned in obesity studies. Nevertheless, one report found *Megasphaera* to be less prevalent in individuals with hypertension^41^, which is associated with obesity as part of the metabolic syndrome.

When we focused in more detail on the microbiome composition on the intra-individual level, we discovered exceptions from these generalized observations. In many cases, we detected a small number of patients in which a given bacterial taxon decreased after surgery, while in the rest of the group it significantly increased, or vice versa. We showed that individual factors determine the resulting bacterial composition much more than the decreasing BMI. A similar observation was reported by a recent study on the gut microbiome which highlighted regional differences in changes of gut microbiome composition after bariatric surgery^42^. Another study, in which gut microbiome was sampled 1, 3 and 12 months after bariatric surgery, also pointed to individual-specific changes of the gut microbiome composition^43^. There are many factors that can influence the salivary microbiome composition of an individual, such as dental hygiene, oral health status, nutritional composition of the food, immunological factors and also the individual-specific resident bacteria which may impede the colonization by new species^44,45^. Many of these factors cannot be quantified precisely, or their action on the salivary microbiome is not straightforward. Therefore, which of them has a greater impact on the salivary microbiome composition than the rapid decrease of body weight, remains an open question.

## Methods

The study was approved by the Ethics Committee of the Břeclav Hospital, Břeclav, Czech Republic and by the Ethics Committee of the Institute of Molecular Biomedicine, Comenius University, Bratislava, Slovakia. The ethical clearance for this study was obtained from The Ethical Committee of Břeclav Hospital. All methods were carried out in accordance with relevant guidelines and regulations.

### Saliva collection and DNA extraction

Unstimulated whole mouth saliva was collected from 35 patients with obesity scheduled for bariatric surgery at the Břeclav Hospital. Samples taken before and 1 day after bariatric surgery were collected at the surgical department and samples taken 3 months and 12 months after surgery were collected at the bariatric outpatients’ department. Oral hygiene was not monitored before collecting the post-surgery samples. An informed consent was obtained from each participant prior to sampling. All patients received general dietary recommendations for bariatric patients including taking small portions more frequently, chewing well and many others as described in the literature^46^. Exclusion criteria included an active systematic inflammatory state of the patient, cancer or heart failure, but none of the patients had to be excluded. At least 2 ml of saliva was collected by passive drooling into sterile tubes. The volunteers were instructed not to eat in the morning before saliva collection. All samples were immediately frozen after collection and stored at − 20 °C until further processing. Samples were thawed at room temperature and centrifuged at 1600×*g* for 10 min. Total DNA was extracted from 200 µl of the supernatants using QIAamp 96 DNA Blood kit (Hilden, Germany) following the protocol for body fluids.

### 16S rRNA gene sequencing

Regions V3 and V4 of the 16S rRNA gene were amplified using a forward primer TCG TCG GCA GCG TCA GAT GTG TAT AAG AGA CAG CCT ACG GGN GGC WGC AG and the reverse primer GTC TCG TGG GCT CGG AGA TGT GTA TAA GAG ACA GGA CTA CHV GGG TAT CTA ATC C^47^ and Kapa HiFi Hot Start polymerase (Ref. 7958935001) as in our previous salivary microbiome study^39^. The PCR conditions were the following: 95 °C for 30 s, 55 °C for 30 s, 72 °C for 30 s repeated in 25 cycles. The samples were multiplexed using 96 index combinations and sequenced on the Illumina platform with MiSeq Reagent Kit v3 (600-cycle, Ref. MS-102-3003).

### Sequence analysis

Sequence data were analyzed using Mothur v1.39.5^48^. Briefly, overlapping pair-end reads were aligned to produce full-length sequences of target region. Reads containing ambiguous bases (N), homopolymer segment (> 8), or reads which were too long (> 470), were discarded. Subsequently, chimeras were identified using the VSEARCH algorithm within Mothur^49^. Resultant reads were clustered to form OTU with 3% cut-off. To determine bacterial composition in each sample the reads were aligned with 80% cut off against Human Oral Microbiome Database v 15.1. The names of OTU in this study were composed from the bacterial species name and the OTU number.

### Statistical analysis

For the first comparison, the samples were grouped by pairs of time-points: (a) before and 1 day after surgery, (b) before surgery and 3 months after surgery, (c) before surgery and a year after surgery, (d) 3 months and 1 year after surgery. The bacterial composition (involving OTUs with an average proportion across the samples > 0.1%) was first analyzed using principal component analysis by comparing different pairs of time-points using the “vegan” R package v2.4^50^. The same package was used for PERMANOVA to assess differences between paired time-points. In addition, the bacterial composition of the paired time-points was compared using the package “edgeR” R package v3.26^51^ with default settings and a significance threshold of p-value < 0.001.

In the next step, all the samples from time-points before surgery, 3 months after the surgery and a year after the surgery were analyzed together for their relation to BMI. In order to compare the influence of the factors of “BMI” and “patients’ individuality” on the ordination of samples in the canonical correspondence analysis, the “envfit” function from “vegan” R package was used. In addition, the proportion of each bacterial OTU was tested for its correlation with BMI using the “Hmisc” R package v4.2^52^ using Spearman correlations. The correlation values > 0.3 and < − 0.3 were then filtered by their p-value at a level of 0.05 for statistical significance. Correlations have been then plotted using R packages “corrplot” v0.84^53^ and “beeswarm” v0.2.3^54^. The same cutoff was used for correlations between all OTUs in all collected samples.

As the last step, the temporal changes of each participant were analyzed separately on intra-individual level. For that purpose, the proportion of each OTU was analyzed by comparing the seven pairs of time-points described above. In order to ease the interpretation of the results, the temporal changes of each OTU in each patient were categorized into 5 groups: increase by > 100% compared to the previous time-point, increase by 5–100% compared to the previous time-point, relatively stable between the given pair of time-points, decrease by 5–100% compared to the previous time-point, decrease by > 100% compared to the previous time-point.

## Supplementary information

Supplementary Information.

## Data Availability

The amplicon reads are available at the Sequence Read Archive under the BioProject PRJNA590249.

## References

[1] Hruby A, Hu FB (2015). The epidemiology of obesity: A big picture. Pharmacoeconomics.

[2] Ng M (2014). Global, regional, and national prevalence of overweight and obesity in children and adults during 1980–2013: A systematic analysis for the Global Burden of Disease Study 2013. Lancet.

[3] Turnbaugh PJ (2006). An obesity-associated gut microbiome with increased capacity for energy harvest. Nature.

[4] Zhao L (2013). The gut microbiota and obesity: From correlation to causality. Nat. Rev. Microbiol..

[5] Fetissov SO (2017). Role of the gut microbiota in host appetite control: Bacterial growth to animal feeding behaviour. Nat. Rev. Endocrinol..

[6] Muscogiuri G (2019). Gut microbiota: A new path to treat obesity. Int. J. Obes. Suppl..

[7] Zeigler CC (2012). Microbiota in the oral subgingival biofilm is associated with obesity in adolescence. Obesity.

[8] Goodson JM (2017). The salivary microbiome is altered in the presence of a high salivary glucose concentration. PLoS ONE.

[9] Janem WF (2017). Salivary inflammatory markers and microbiome in normoglycemic lean and obese children compared to obese children with type 2 diabetes. PLoS ONE.

[10] Mervish NA (2019). Associations of the oral microbiota with obesity and menarche in inner city girls. J. Child. Obes..

[11] Raju SC (2019). Gender-specific associations between saliva microbiota and body size. Front. Microbiol..

[12] Wang RR (2019). Association of the oral microbiome with the progression of impaired fasting glucose in a Chinese elderly population. J. Oral. Microbiol..

[13] Dalile B, Van Oudenhove L, Vervliet B, Verbeke K (2019). The role of short-chain fatty acids in microbiota–gut–brain communication. Nat. Rev. Gastroenterol. Hepatol..

[14] Hoffman KL (2018). Oral microbiota reveals signs of acculturation in Mexican American women. PLoS ONE.

[15] Wu Y, Chi X, Zhang Q, Chen F, Deng X (2018). Characterization of the salivary microbiome in people with obesity. PeerJ.

[16] Duvallet C, Gibbons SM, Gurry T, Irizarry RA, Alm EJ (2017). Meta-analysis of gut microbiome studies identifies disease-specific and shared responses. Nat. Commun..

[17] Gibbons SM (2019). Defining microbiome health through a host lens. mSystems.

[18] Adams TD (2017). Weight and metabolic outcomes 12 years after gastric bypass. N. Engl. J. Med..

[19] Pories WJ (2008). Bariatric surgery: Risks and rewards. J. Clin. Endocrinol. Metab..

[20] Schauer PR, Nor Hanipah Z, Rubino F (2017). Metabolic surgery for treating type 2 diabetes mellitus: Now supported by the world's leading diabetes organizations. Cleve Clin. J. Med..

[21] Damms-Machado A (2015). Effects of surgical and dietary weight loss therapy for obesity on gut microbiota composition and nutrient absorption. Biomed. Res. Int..

[22] Tremaroli V (2015). Roux-en-Y gastric bypass and vertical banded gastroplasty induce long-term changes on the human gut microbiome contributing to fat mass regulation. Cell Metab..

[23] Ilhan ZE (2017). Distinctive microbiomes and metabolites linked with weight loss after gastric bypass, but not gastric banding. ISME J..

[24] Elliott JA, Reynolds JV, le Roux CW, Docherty NG (2016). Physiology, pathophysiology and therapeutic implications of enteroendocrine control of food intake. Expert Rev. Endocrinol. Metab..

[25] Sweeney TE, Morton JM (2014). Metabolic surgery: Action via hormonal milieu changes, changes in bile acids or gut microbiota? A summary of the literature. Best Pract. Res. Clin. Gastroenterol..

[26] Paganelli FL (2019). Roux-Y gastric bypass and sleeve gastrectomy directly change gut microbiota composition independent of surgery type. Sci. Rep..

[27] Li JV (2011). Metabolic surgery profoundly influences gut microbial-host metabolic cross-talk. Gut.

[28] Davies NK, O'Sullivan JM, Plank LD, Murphy R (2019). Altered gut microbiome after bariatric surgery and its association with metabolic benefits: A systematic review. Surg. Obes. Relat. Dis..

[29] Guo Y (2018). Modulation of the gut microbiome: A systematic review of the effect of bariatric surgery. Eur. J. Endocrinol..

[30] Shillitoe E (2012). The oral microflora in obesity and type-2 diabetes. J. Oral Microbiol..

[31] Marsicano JA (2011). Interfaces between bariatric surgery and oral health: A longitudinal survey. Acta Cir. Bras..

[32] Hashizume LN (2015). Impact of bariatric surgery on the saliva of patients with morbid obesity. Obes. Surg..

[33] El-Hadi M, Birch DW, Gill RS, Karmali S (2014). The effect of bariatric surgery on gastroesophageal reflux disease. Can. J. Surg..

[34] Sujatha S (2016). Oral pH in gastroesophageal reflux disease. Indian J. Gastroenterol..

[35] Zhou J (2017). Influences of pH and iron concentration on the salivary microbiome in individual humans with and without caries. Appl. Environ. Microbiol..

[36] Cattaneo C (2019). New insights into the relationship between taste perception and oral microbiota composition. Sci. Rep..

[37] Cabral DJ (2017). The salivary microbiome is consistent between subjects and resistant to impacts of short-term hospitalization. Sci. Rep..

[38] Lloyd-Price J (2017). Strains, functions and dynamics in the expanded human microbiome project. Nature.

[39] Džunková M (2018). Oxidative stress in the oral cavity is driven by individual-specific bacterial communities. NPJ Biofilms Microbiomes.

[40] Hall MW (2017). Inter-personal diversity and temporal dynamics of dental, tongue, and salivary microbiota in the healthy oral cavity. NPJ Biofilms Microbiomes.

[41] Yan Q (2017). Alterations of the gut microbiome in hypertension. Front. Cell. Infect. Microbiol..

[42] Medina DA (2019). Cross-regional view of functional and taxonomic microbiota composition in obesity and post-obesity treatment shows country specific microbial contribution. Front. Microbiol..

[43] Aron-Wisnewsky J (2019). Major microbiota dysbiosis in severe obesity: Fate after bariatric surgery. Gut.

[44] Zaura E, Nicu EA, Krom BP, Keijser BJF (2014). Acquiring and maintaining a normal oral microbiome: Current perspective. Front. Cell. Infect. Microbiol..

[45] Acharya A (2017). Salivary microbiome in non-oral disease: A summary of evidence and commentary. Arch. Oral Biol..

[46] Dagan SS (2017). Nutritional recommendations for adult bariatric surgery patients: Clinical practice. Adv. Nutr..

[47] Klindworth A (2013). Evaluation of general 16S ribosomal RNA gene PCR primers for classical and next-generation sequencing-based diversity studies. Nucleic Acids Res..

[48] Schloss PD (2009). Introducing mothur: Open-source, platform-independent, community-supported software for describing and comparing microbial communities. Appl. Environ. Microbiol..

[49] Rognes T, Flouri T, Nichols B, Quince C, Mahé F (2016). VSEARCH: A versatile open source tool for metagenomics. PeerJ.

[50] Oksanen, J. *et al*. *Vegan: Community Ecology Package. R Package Version 2.4.* (2017).

[51] Robinson MD, McCarthy DJ, Smyth GK (2010). edgeR: A bioconductor package for differential expression analysis of digital gene expression data. Bioinformatics.

[52] Harrell, F. E. *Hmisc: Harrell Miscellaneous. R Package Version 4* (2019).

[53] Wei, T. & Simko, V. *Corrplot: Visualization of a Correlation Matrix. R Package Version 0.84* (2018).

[54] Eklund, A. *Beeswarm: The Bee Swarm Plot, an Alternative to Stripchart. R Package Version 0.2.3.* (2016).

