# Supplementary material for: Salivary microbiome composition changes after bariatric surgery
Source: Sci Rep. 2020 Nov 18;10:20086. doi: 10.1038/s41598-020-76991-6 (PMC7674438; doi:10.1038/s41598-020-76991-6)
Supplement: Supplementary file 1 — Supplementary Information. [file 41598_2020_76991_MOESM1_ESM.pdf]

Supplementary Materials for:

Salivary microbiome composition changes after bariatric surgery

Authors:

Mária Džunková[1], Robert Lipták[2], Barbora Vlková[2], Roman Gardlík[2], Michal Čierny[3], Andrés Moya[4,5,6], Peter Celec[2]

1. Department of Energy Joint Genome Institute, Lawrence Berkeley National Laboratory, Berkeley, California, USA
2. Institute of Molecular Biomedicine, Faculty of Medicine, Comenius University, Bratislava, Slovakia
3. Department of Bariatric Surgery, Břeclav Hospital, Břeclav, Czech Republic
4. Department of Genomics and Health, Foundation for the Promotion of Health and Biomedical Research of Valencia Region (FISABIO-Public Health), Valencia, Spain
5. CIBER in Epidemiology and Public Health (CIBEResp), Madrid, Spain
6. Institute for Integrative Systems Biology (I2SysBio), The University of Valencia and The Spanish National Research Council (CSIC)-UVEG, Valencia, Spain

Content:

Supplementary Figure S1: Salivary microbiome composition of all study participants.

Supplementary Table S1: Metadata associated with the study participants.

Supplementary Figure S1: Salivary microbiome composition of all study participants

Salivary microbiome composition of the 35 study participants, each sampled in four time-points: before surgery (1), after surgery (2), three months after surgery (3), twelve months after surgery (4).

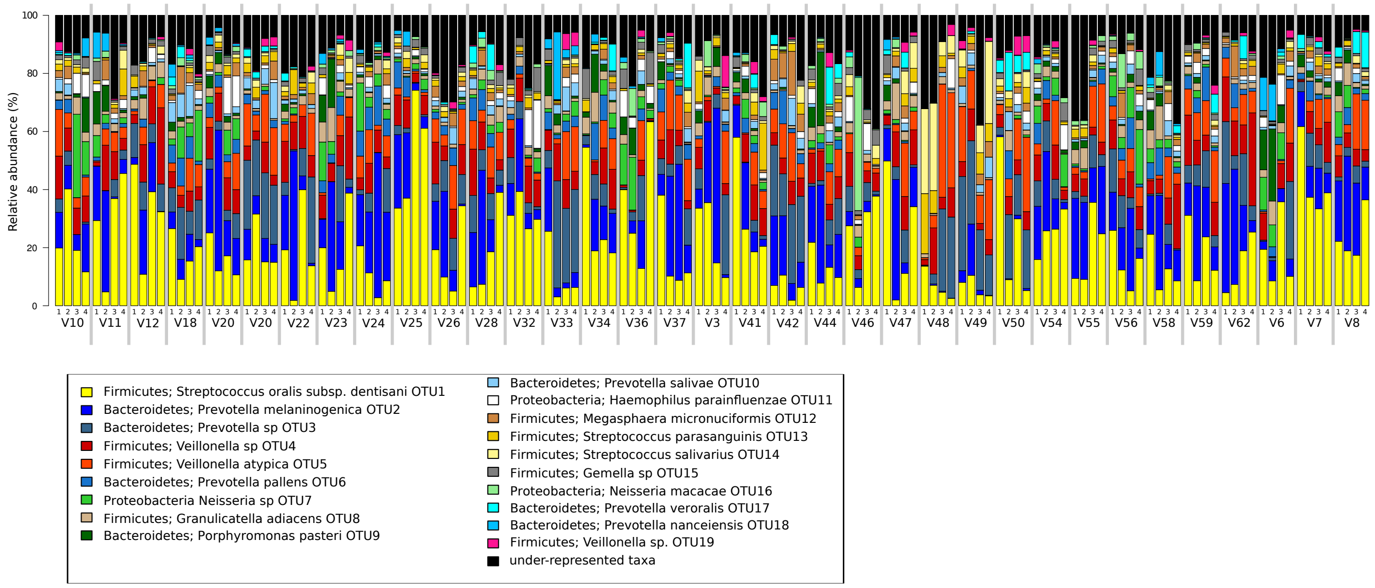

Supplementary Table S1: Metadata associated with the study participants

Patient ID	BMI [kg/m^2] before surgery	BMI [kg/m^2] three months after surgery	BMI [kg/m^2] twelve months after surgery	Sex	Age	Surgery
v10	43.58	37.93	32.28	M	61	Omega Loop Gastric Bypass
v11	43.58	38.79	37.20	M	46	Omega Loop Gastric Bypass
v12	74.22	66.02	54.30	F	52	Sleeve Gastrectomy
v18	41.91	36.20	31.25	F	54	Laparoscopic Gastric Plication
v20	37.87	33.14	32.19	M	47	Laparoscopic Gastric Plication
v21	49.13	44.98	34.95	F	54	Omega Loop Gastric Bypass
v22	51.76	46.40	43.24	M	55	Omega Loop Gastric Bypass
v23	47.33	39.44	35.14	M	48	Omega Loop Gastric Bypass
v24	39.41	35.32	35.69	F	41	Laparoscopic Gastric Plication
v25	42.61	38.31	36.33	F	45	Laparoscopic Gastric Plication
v26	43.21	37.96	33.02	M	60	Omega Loop Gastric Bypass
v28	41.91	37.18	33.80	F	38	Laparoscopic Gastric Plication
v3	40.12	34.26	31.17	M	40	Laparoscopic Gastric Plication
v32	38.10	33.15	33.53	F	44	Laparoscopic Gastric Plication
v33	38.86	32.56	32.56	F	50	Laparoscopic Gastric Plication
v34	36.68	30.80	30.10	F	48	Laparoscopic Gastric Plication
v36	38.61	34.03	33.74	M	57	Laparoscopic Gastric Plication
v37	46.68	40.51	33.18	F	52	Roux-en-Y Gastric Bypass
v41	48.48	43.71	43.34	F	33	Laparoscopic Gastric Plication
v42	38.62	35.43	31.18	F	58	Laparoscopic Gastric Plication
v44	54.53	44.73	43.76	M	30	Laparoscopic Gastric Plication
v46	53.15	43.58	44.29	M	32	Laparoscopic Gastric Plication
v47	54.33	46.40	39.38	M	39	Sleeve Gastrectomy
v48	40.14	34.60	28.72	F	58	Roux-en-Y Gastric Bypass
v49	38.12	27.38	31.41	M	45	Laparoscopic Gastric Plication
v50				F	33	Roux-en-Y Gastric Bypass
v54	42.37	36.76	36.06	F	36	Laparoscopic Gastric Plication
v55	48.67	39.71	31.05	M	42	Laparoscopic Gastric Plication
v56	40.22	35.49	35.83	M	63	Laparoscopic Gastric Plication
v58	43.83	37.98	35.94	M	59	Sleeve Gastrectomy
v59	41.62	36.13	35.26	M	47	Roux-en-Y Gastric Bypass
v6	49.08	38.67	31.98	F	52	Roux-en-Y Gastric Bypass
v62	36.81	33.46	33.46	F	60	Laparoscopic Gastric Plication
v7	44.92	40.96	38.97	M	37	Omega Loop Gastric Bypass
v8	59.34	51.45	47.34	M	60	Laparoscopic Gastric Plication
